# Prevalence and mortality rate of abdominal surgical emergencies in Sub-Saharan Africa: a systematic review and meta-analysis

**DOI:** 10.1186/s12893-024-02319-0

**Published:** 2024-01-24

**Authors:** Abdourahmane Ndong, Lebem Togtoga, Mamadou Saïdou Bah, Papa Djibril Ndoye, Khadim Niang

**Affiliations:** 1 Department of Public Health and Social Medicine, Faculty of Health Sciences, Gaston Berger University, Saint-Louis, Senegal; 2 General Surgery Department, Regional Hospital Center of Saint Saint-Louis, Saint-Louis, Senegal

**Keywords:** Emergency, Abdomen, Africa, Mortality, Surgery

## Abstract

**Introduction:**

Abdominal surgical emergencies remain prevalent in various healthcare settings, particularly in regions with limited access to basic surgical care, such as Africa. The aim of this literature review is to systematically assess publications on abdominal surgical emergencies in adults in sub-Saharan Africa to estimate their prevalence and mortality rate.

**Methodology:**

A systematic review was conducted. The latest search was performed on October 31, 2022. We estimated the pooled prevalence with a 95% confidence interval (CI) for each abdominal surgical emergency, as well as overall postoperative mortality and morbidity rates.

**Results:**

A total of 78 studies were included, and 55.1% were single-center retrospective and monocentric studies. The mean age of the patients was 32.5 years, with a sex ratio of 1.94. The prevalence of each abdominal surgical emergency among all of them was as follows: appendicitis: 30.0% (95% CI: 26.1–33.9); bowel obstruction: 28.6% (95% CI: 25.3–31.8); peritonitis: 26.6% (95% CI: 22.2–30.9); strangulated hernias: 13,4% (95% CI: 10,3–16,5) and abdominal trauma: 9.4% (95% CI: 7.5–11.3). The prevalence of complications was as follows: mortality rate: 7.4% (95% CI: 6.0-8.8); overall postoperative morbidity: 24.2% (95% CI: 19.4–29.0); and surgical site infection 14.4% (95% CI: 10.86–18.06).

**Conclusion:**

Our study revealed a high prevalence of postoperative complications associated with abdominal surgical emergencies in sub-Saharan Africa. More research and efforts should be made to improve access and quality of patient care.

## Introduction

Emergency surgery refers to surgical procedures that cannot be safely postponed without adversely affecting the patient’s clinical condition. It is typically conducted urgently, often within a brief period after the patient’s admission to the hospital, usually within hours. This situation presents a distinctive context compared to elective surgery, as patients undergoing emergency procedures require special monitoring due to their lack of preparation and the physiological disorders that can further compromise them in addition to the surgical stress [1].

Abdominal surgical emergencies remain frequent in practice regardless of the context. Africa represents one of the regions most affected by the lack of access to basic surgical care, particularly in emergency settings. In fact, it is estimated that 93% of its population does not have access to emergency surgery when they need it [1].

This represents a significant burden in addition to other public health emergencies, such as HIV, tuberculosis, and malaria. Abdominal surgical emergencies accounted for a large proportion of surgeries performed in African hospitals, with a frequency varying between 20 and 22.7% [2, 3].

Patients with abdominal surgical emergencies, when compared to those operated on in a scheduled setting, have their risk of death multiplied up to five times [4]. The factors that can explain this higher rate of complications in emergency surgery are: the lack of patient preparation in the context of urgency, acute physiological disorders caused by urgent pathologies such as sepsis, hypovolemia, and hydro-electrolytic disorders. Additionally, comorbid conditions that may be present in emergency patients are often not optimized, increasing the anesthetic risk of postoperative complications [4].

Additionally, these deaths remain higher in developing countries, ranging from 4.9 to 13.2% [4, 5, 9].

Furthermore, there is a significant lack of data in Sub-Saharan Africa. Indeed, the majority of these countries do not have national registries or audit systems to monitor surgical care and associated complications [6].

To improve the quality of care for patients and tailor risk assessment for our specific context, there is an urgent need for quality data on this topic [7]. This will address the limited understanding of the burden of abdominal surgical emergencies and help identify areas for improvement .

To better understand this topic, we performed a systematic review to assess publications on abdominal surgical emergencies in adults in sub-Saharan Africa to estimate their prevalence and mortality rate.

## Methodology

We conducted a systematic review to study abdominal surgical emergencies in adults in Sub-Saharan Africa.

The main objectives were to:

- Determine the prevalence of each abdominal surgical emergency (appendicitis, bowel obstruction, peritonitis, strangulated hernias and abdominal traumas) in Sub-Saharan Africa;
- Determine the pooled prevalence of postoperative mortality and overall morbidity associated with abdominal surgical emergencies in Sub-Saharan Africa.

### Research strategy

The protocol of this systematic review was registered at https://www.researchregistry.com/ (reviewregistry1771). Two individuals, AN and LT, jointly conducted the study search, inclusion, and data extraction.

This review followed the PRISMA (Preferred Reporting Items for Systematic Reviews and Meta-Analyses) guidelines [27]. An extensive search was performed on the following online databases:

- Pub Med/Medline and;
- African Journal Online.

The keywords used in the search process included the following:

- “emergency general surgery OR emergency laparotomy” OR “emergency laparotomy OR emergency general surgery,”
- “Abdomen, Acute/surgery“[MeSH] and;
- “Africa South of the Sahara“[MeSH].

The search was carried out using various combinations of these terms. Additionally, a supplementary manual search was conducted on the following search engines: *ResearchGate* and *Google Scholar*. Furthermore, we checked the references of all included articles to find relevant publications. The latest search was conducted on October 31, 2022.

The retrieved references were managed using Rayyan software [28]. Zotero software was used to download the references, remove duplicates, and perform primary and secondary selection analyses.

### Inclusion criteria

Using the PICOS framework (Patient, Intervention, Comparison, Results, Study type), the inclusion criteria were as follows:

- Patients: Adults (over 15 years) with abdominal surgical emergencies (appendicitis, acute intestinal obstruction, peritonitis, strangulated hernias and abdominal traumas);
- Interventions: Surgical treatment;
- Comparison: None;
- Outcomes: Prevalence of each emergency, prevalence of mortality, and overall morbidity;
- Study Type: Observational, randomized or nonrandomized, prospective or retrospective studies with more than 20 patients published in English or French.

### Exclusion criteria

We excluded studies that described the following:

- Only obstetric emergencies;
- Only postoperative complications;
- Only imaging or anesthesia data;
- Only patients operated on with laparoscopy;
- Only one surgical emergency;
- Mixed cohorts with other surgical emergencies where data on abdominal emergencies could not be extracted;
- Mixed cohorts with elective and emergency surgery where data on abdominal emergencies could not be extracted.

Letters to editors, literature reviews, and duplicated studies were also excluded.

### Data extraction

The extracted data included study type, year of publication, country, total number of patients, age, sex, number of each surgical emergency, surgical site infection and mortality and overall morbidity rates.

For data extraction of postoperative outcomes, we considered the following:

- Surgical site infection: infection at the abdominal wound during the postoperative period, whether superficial or deep;
- Overall morbidity: the total number of complications (of any type) among the total number of patients;
- Mortality: the number of reported deaths among the total number of patients in the study, regardless of the evaluation period (at 30 days, 90 days, during hospitalization, or unspecified).

### Quality assessment

The Newcastle‒Ottawa Scale (NOS) (selection criteria and outcome criteria) was used to assess the quality of the included studies [8–11].

- A score of 6 was considered good quality.
- A score of 4 or 5 as moderate quality; and.
- A score of 3 or less was considered poor quality.

### Risk of bias assessment

A funnel plot was used to depict publication bias using Jamovi software Version 2.4.1.0. A rank correlation test with Kendall’s tau statistic was employed to detect asymmetry when *p* ≤ 0.05, indicating a publication bias.

### Data analysis and statistical methods

Statistical analysis was performed using R Studio software Version 1.2.5042. Graphs and maps were created using Microsoft Excel. For qualitative variables, the number with their proportions were described. For quantitative variables, the mean and standard deviation were used.

A meta-analysis was conducted to estimate the combined prevalence with a 95% confidence interval (CI) for each abdominal surgical emergency and the overall mortality and morbidity rates in Sub-Saharan Africa.

Heterogeneity among the studies was tested using the I^2^ test. A random-effects model was used when I^2^ > 50%, and a fixed-effects model was used when I^2^ ≤ 50%.

## Results

**Fig. 1:**
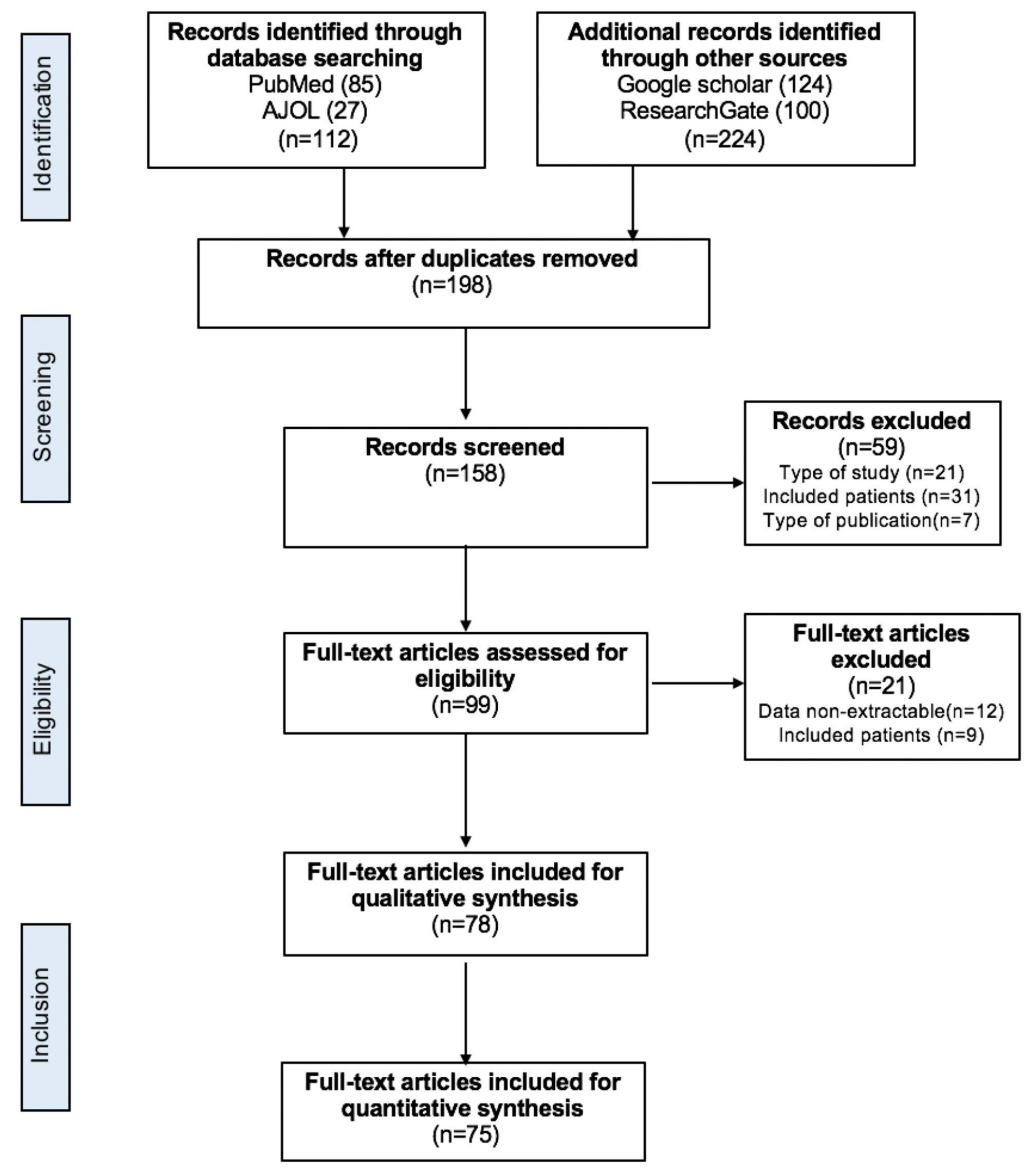
PRISMA flow diagram illustrating the search process and study selection

We found 112 articles in the databases and 224 through manual internet searches. After removing duplicates, 198 articles were evaluated. We identified 79 studies on abdominal surgical emergencies over the explored period of 32 years (1981–2022). After excluding articles that did not meet the inclusion criteria, we had 78 articles for the qualitative synthesis, of which 75 were included in the quantitative synthesis. The PRISMA flow chart is illustrated in Fig. 1.

### Study characteristics

In total, there were 38,187 patients with a range of 48 to 3,717 patients per study. The mean age of the patients was 32.5 years, with a range of 22.9 to 47 years. The sex ratio was 1.94, with 65.9% male (*n* = 17,656) and 34.1% female (*n* = 9,097). Most studies were retrospective (55.1%, *n* = 43). The quality of the studies was judged as good in 12.8% (*n* = 10), moderate in 50% (*n* = 39), and low in 37.2% (*n* = 29).

Table 1 and Table 2 summarize the characteristics of the different studies used for the literature review.

**Table 1:** Characteristics of studies included in the systematic review (*n* = 78) (A)

Characteristics	Mean (extrems) Number(percentage)	Number of studies with available data
Number of patients	38,187 (48-3717)	78/78
Average age	32.5 (22.9–47)	65/78
Sex ratio	1.94	64/78
Female	9097	
Male	17,656	
Type of study		75/78
Retrospective	43 (55.1)	
Prospective	32 (41)	
NA (not available)	3 (3.9)	
Study quality (NOS scale)		78/78
Good	10 (12.8)	
Moderate	39 (50)	
Poor	29 (37.2)	
Year of publication		78/78
Before 2000	2 (2.8)	
2000–2004	3 (3.8)	
2005–2009	9 (11.5)	
2010–2014	9 (11.5)	
2015–2019	28 (35.8)	
After 2020	27 (34.6)	
Study location		78/78
West Africa	42 (53.8)	
Central Africa	11 (14.1)	
East Africa	17 (28.8)	
Southern Africa	3 (3.8)	

**Table 2:** Characteristics of studies included in the systematic review (*n* = 78) (B)

Study	Year	Country	Type of study	N(total)	Mean Age (years)	Male	Female
Adamu [12]	2010	Nigeria	Prospective	488	32	301	187
Ademe [13]	2022	Ethiopia	Retrospective	267	36	160	107
Agboola [14]	2014	Nigeria	Prospective	276	NA	197	79
Ahmed [15]	2010	Nigeria	Prospective	3717	32.5	NA	NA
Ajao [16]	1981	Nigeria	NA	360	NA	240	120
Almeimoune [17]	2021	Mali	Prospective	631	36.13	NA	NA
Assouto [18]	2009	Benin	Retrospective	613	30	377	236
Attipou [19]	2005	Togo	Retrospective	932	32	600	332
Awori [20]	2005	Kenya	Prospective	398	NA	NA	NA
Ayenew [21]	2016	Ethiopia	Retrospective	295	33.7	230	65
Ba [22]	2021	Senegal	Prospective	601	30.2	428	173
Bang [23]	2021	Cameroon	Prospective	120	37.6	80	40
Camara [24]	2021	Guinea Conakry	Retrospective	460	41.5	297	163
Coulibaly [25]	2019	Mali	Prospective	100	34.4	70	30
Daddy [26]	2020	Niger	Prospective	262	26.57	190	72
Debrah [27]	2012	Ghana	Retrospective	122	31.3	63	59
Dembélé [28]	2021	Mali	Prospective	101	33.6	89	12
Dewulf [29]	1986	Rwanda	NA	204	NA	NA	NA
Diallo [30]	2020	Gabon	Retrospective	311	29.5	177	34
Diaw [31]	2018	Senegal	Retrospective	90	34	64	26
Didier [32]	2020	Niger	Prospective	151	25.2	106	45
Diop [33]	2011	Senegal	Retrospective	504	39.6	343	161
Dossouvi [34]	2021	Togo	Retrospective	204	29	125	79
Doui [35]	2009	Central Africa	Prospective	160	35.6	108	52
Doumi [36]	2009	Sudan	Prospective	421	NA	242	179
Ehlers [9]	2021	South Africa	Retrospective	3609	NA	NA	NA
Engbang [66]	2021	Cameroon	Prospective	218	36.32	112	91
Gaye [38]	2016	Senegal	Retrospective	161	41	120	41
Gbessi [39]	2015	Benin	Retrospective	169	NA	96	73
Gebre [40]	2016	Ethiopia	Retrospective	166	27.2	94	72
Gebrie [41]	2019	Ethiopia	Prospective	192	31.46	107	64
Hagos [42]	2015	Ethiopia	Retrospective	299	31.5	240	59
Hanks [43]	2014	Ethiopia	Retrospective	328	35.6	NA	NA
Harissou [44]	2015	Niger	Prospective	302	23	227	75
Harouna [45]	2001	Niger	Retrospective	742	NA	NA	NA
Ibrahim [46]	2015	Nigeria	Prospective	612	44.9	NA	NA
Kambire [47]	2017	Burkina	Retrospective	343	29	265	78
Kambire [48]	2018	Burkina	Retrospective	394	33	290	104
Karuhanga [49]	2020	Tanzania	Retrospective	284	39	185	99
Kassegne [50]	2015	Togo	Retrospective	303	24	242	61
Katswere [51]	2018	Benin	Prospective	128	30	81	47
Korsé [52]	2021	Guinea Conakry	Prospective	135	34	93	42
Kotiso [53]	2007	Ethiopia	Retrospective	587	30.7	391	196
Madubogwu [54]	2020	Niger	Retrospective	177	33.98	92	85
Magagi [2]	2016	Niger	Prospective	622	22.9	467	155
Mbah [55]	2006	Nigeria	Prospective	136	25	95	41
Mcconkey [56]	2022	Sierra Leone	Retrospective	173	NA	NA	NA
Melkie [57]	2016	Ethiopia	Retrospective	304	NA	188	183
Mjema [58]	2020	Tanzania	Prospective	199	47	73	126
Motto [59]	2021	Cameroon	Prospective	63	41.06	NA	NA
Mpirimbanyi [60]	2020	Rwanda	Retrospective	563	28	377	186
Mpirimbanyi [61]	2017	Rwanda	Retrospective	51	NA	NA	NA
Nega [62]	2009	Ethiopia	NA	143	26.6	79	64
Negash [63]	2017	Ethiopia	Retrospective	238	26.5	196	74
Ngakani [64]	2020	Gabon	Retrospective	451	35.3	282	169
Nyundo [65]	2013	Rwanda	Prospective	229	28.8	144	85
Obonna [66]	2014	Nigeria	Retrospective	2408	35	1605	803
Ogbuanya [67]	2021	Nigeria	Retrospective	879	NA	NA	NA
Ogbuanya [67]	2016	Nigeria	Prospective	684	38.89	398	286
Ohene-yeboah [68]	2006	Ghana	Prospective	3114	32.8	2040	1074
Paluku [69]	2018	Benin	Prospective	128	NA	81	47
Paluku [70]	2020	Benin	Prospective	76	28	43	33
Rahman [71]	2018	Ghana	Retrospective	411	36.3	287	124
Sanogo [72]	2020	Mali	Retrospective	334	24	114	220
Smith [73]	2021	South Africa	Retrospective	1464	34	861	603
Songne [74]	2008	Togo	Retrospective	943	42	697	246
Soumah [75]	2011	Senegal	Retrospective	88	23.19	58	30
Spence [76]	2016	South Africa	Prospective	169	34.9	116	53
Tamegnon [77]	2021	Togo	Retrospective	219	29	140	79
Tassew [78]	2017	Ethiopia	Retrospective	299	33.9	211	98
Tendeng [79]	2018	Senegal	Prospective	118	35.9	94	24
Tounkara [80]	2018	Mali	Prospective	120	30	77	43
Tsegaye [81]	2006	Ethiopia	Retrospective	511	32	389	122
Valimungighe [82]	2015	Congo	Prospective	203	30.2	82	121
Wossen [83]	2019	Ethiopia	Retrospective	439	28.4	332	107
Yawo [84]	2021	Guinea Conakry	Retrospective	412	31.36	218	194
Zare [85]	2020	Burkina	Retrospective	675	36	498	177
Zare [86]	2018	Burkina	Retrospective	426	30	314	112

**Fig. 2:**
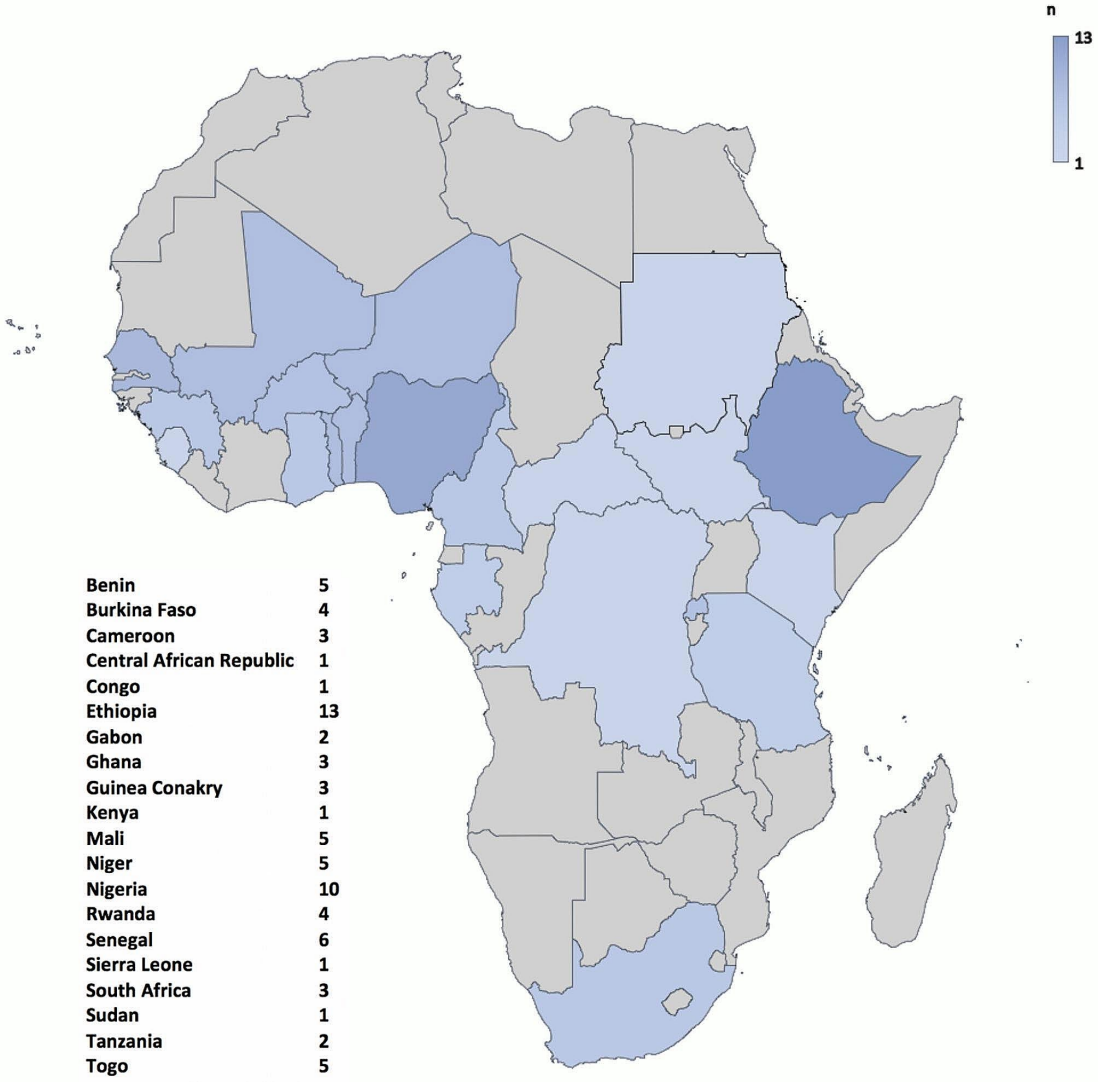
Map of Sub-Saharan Africa showing the number of studies on abdominal surgical emergencies per country (*n* = 78)

Studies were found in 20 countries. The majority of studies originated from West Africa (53.8%, *n* = 42), followed by East Africa (28.8%, *n* = 17), Central Africa (14.1%, *n* = 11), and Southern Africa (3.8%, *n* = 3) (Fig. 2).

There was a progressive increase in the number of studies published over the years, with 55 studies (70.5%) published after 2015.

**Fig. 3:**
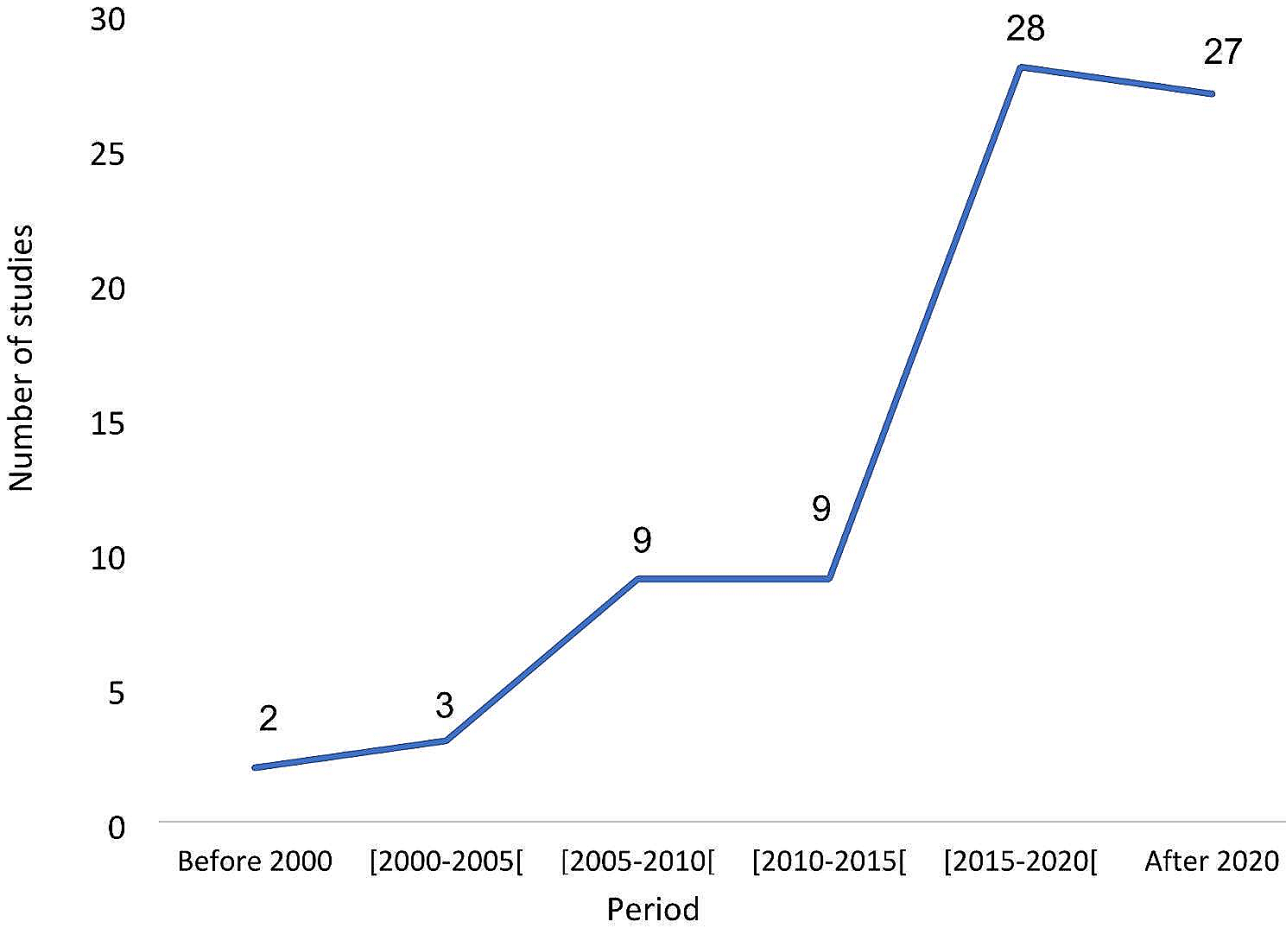
Trend in the number of publications on abdominal surgical emergencies in Sub-Saharan Africa (*n* = 78)

Figure 3 represents the trend in the number of publications on abdominal surgical emergencies in Sub-Saharan Africa over the last 32 years.

### Meta-analysis

**Fig. 4:**
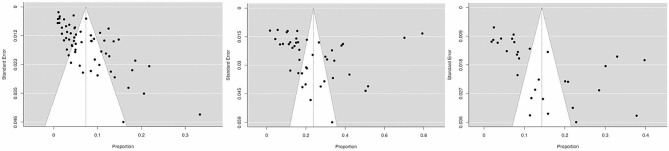
Funnel plots for assessment of publication bias in the three study outcomes in the meta-analysis. **A**: Mortality (tau = 0.343; *p* < 0.001); **B**: Overall morbidity (tau = 0.350; *p* < 0.001); **C**: Surgical site infection (tau = 0.461; *p* < 0.001).

The heterogeneity was high accross all studies, and a random effect model was used for the meta-analysis. There was a high publication bias for the meta-analysis of outcomes, as represented in the funnel plot at Fig. 4.

The results of the meta-analysis estimating the pooled prevalence of each abdominal surgical emergency and the postoperative complications are detailed in Table 3.

**Table 3:** Meta-analysis estimating the pooled prevalence of each abdominal surgical emergency and postoperative complications

Meta-analysis of prevalence	Pooled prevalence	95% Confidence interval		I^2^ test of heterogeneity	Model
**Abdominal surgical emergency**					
Appendicitis	30	26.1	33.9	98.8%	Random
Bowel obstruction	28.6	25.3	31.8	98.3%	Random
Peritonitis	26.5	22.2	30.9	99.0%	Random
Strangulated hernias	13.4	10.3	16.5	97.1%	Random
Abdominal traumas	9.4	7.5	11.3	95.9%	Random
**Complications**					
Mortality	7.4	6.0	8.8	94.5%	Random
Overall morbidity	24.2	19.4	29.0	99.0%	Random
Surgical site infection	14.4	10.8	18.0	97.5%	Random

The prevalence of each cause among abdominal surgical emergencies in Sub-Saharan Africa was as follows:

- Appendicitis: 30.0% (95% CI: 26.1–33.9);
- Acute intestinal obstruction: 28.6% (95% CI: 25.3–31.8);
- Peritonitis: 26.5% (95% CI: 22.2–30.9);
- Strangulated hernias: 13,4% (95% CI: 10,3–16,5);
- Abdominal traumas: 9.4% (95% CI: 7.5–11.3).

The prevalence of complications associated with abdominal surgical emergencies was as follows:

- Mortality: 7.4% (95% CI: 6.0-8.8);
- Overall postoperative morbidity: 24.2% (95% CI: 19.4–29.0);
- Postoperative infections: 14.4% (95% CI: 10.8–18.0).

**Fig. 5:**
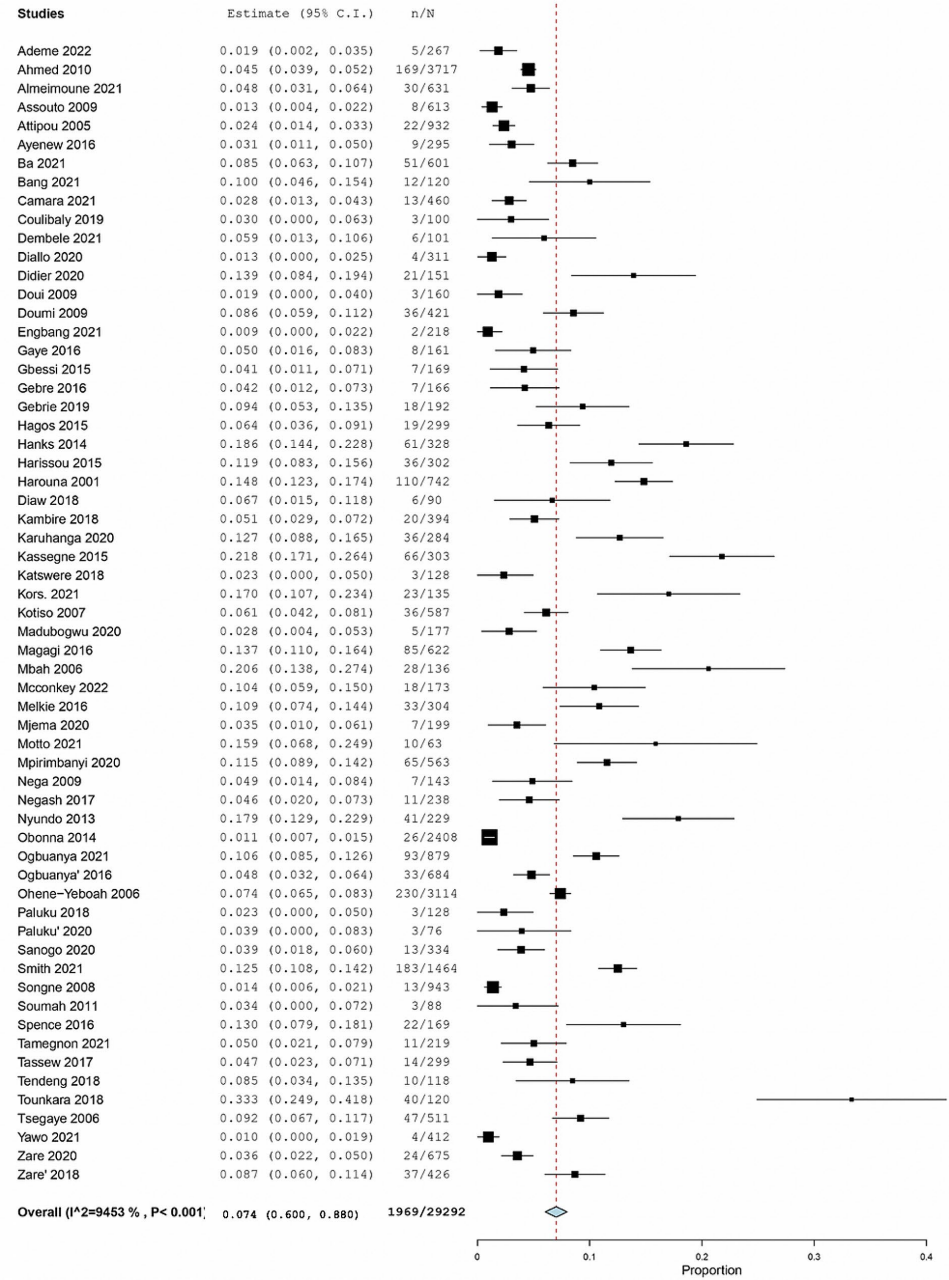
Forest plot estimating the pooled prevalence of overall mortality

**Fig. 6:**
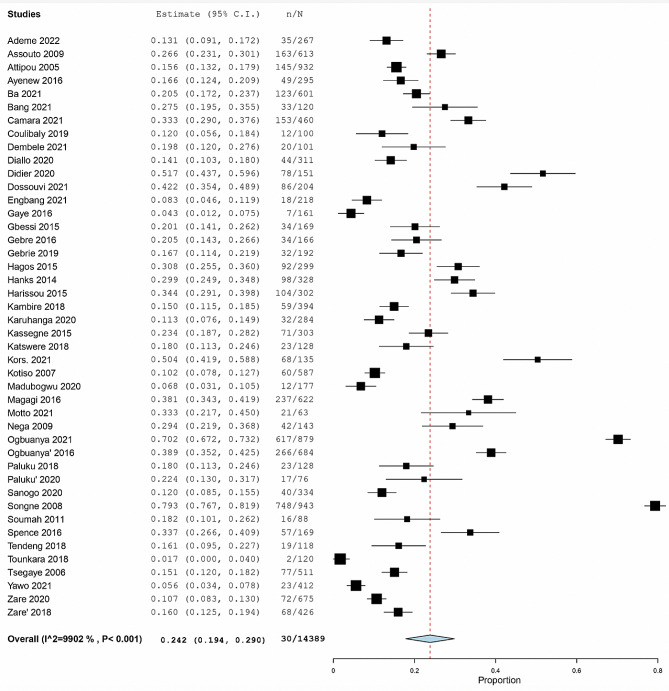
Forest plot estimating the pooled prevalence of overall morbidity

**Fig. 7:**
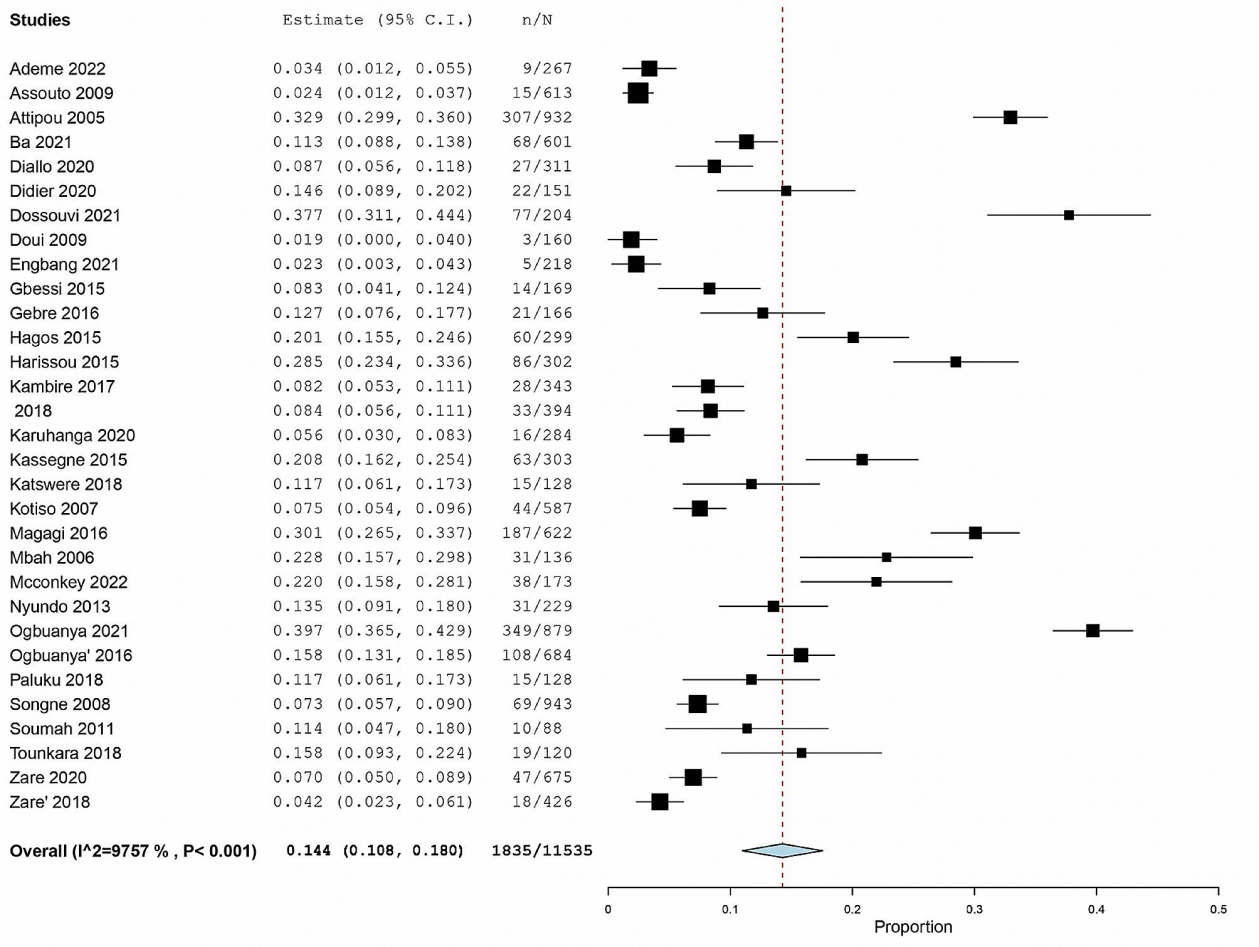
Forest plot estimating the pooled prevalence of surgical site infection

The Figs. 5, 6 and 7 represent the forest plots estimating the pooled prevalence of post operatives complications.

## Discussion

Several research studies on abdominal surgical emergencies in Africa have been conducted; however, the data have often been reported in specific settings. The aim of this review was to assess the prevalence of each surgical emergency among all causes of abdominal surgical emergencies and to determine the prevalence of postoperative complications in Sub-Saharan Africa. This review included 78 research studies published over a 32-year period involving 38,187 patients.

In our literature review, acute appendicitis was the leading cause of abdominal surgical emergencies in Sub-Saharan Africa, with a combined prevalence of 30.0% (95% CI: 26.1–33.9). Our review encompasses 112 articles from 20 countries in sub-Saharan Africa. The majority of studies on this topic were conducted within a single country or institution. This shows how our pooled data appears to be representative, providing insight into the prevalence of appendicitis in sub-saharan Africa. Globally, it is the most frequent cause of digestive surgical emergencies. Its prevalence ranges from approximately 44.2% and 62.8% depending on countries [87, 88]. For a condition with well-codified treatment and not requiring a significant amount of resources, the associated mortality remains high. It was estimated in 2019 that out of 17.7 million cases worldwide, there were 33,400 deaths [89]. In addition, existing data suggest a mortality rate of 54 per 1000 appendectomies in Sub-Saharan Africa, compared to 3.03 per 1000 appendectomies in developed countries [90]. This significant difference is mainly attributed to difficulties in accessing surgical care and diagnostic delays.

Our review revealed that acute intestinal obstruction had a prevalence of 28.6% (95% CI: 25.3–31.8) among abdominal surgical emergencies in Sub-Saharan Africa. It constitutes the leading cause of surgical emergencies in different studies, accounting for 43.2% in some studies [79].

The causes of intestinal obstructions differ in frequency depending on the geographical area. In developed countries, obstruction due to adhesions and tumors are predominant, while colonic volvulus is more prevalent in Sub-Saharan Africa [2, 91, 92]. Associated mortality reached 9.2% in certain series in Africa, mainly due to long delays in seeking medical care and the occurrence of bowel necrosis [91].

Strangulated hernias had a pooled prevalence of 13,4% (95% CI: 10,3–16,5). With this important rate of emergency surgery for hernia, efforts should be made to improve the availability and accessibility of surgery for the entire population [93]. This will help to prevent the occurrence of strangulation and reduce the risk of postoperative complications. A recent literature review on inguinal hernias in Sub-Saharan Africa showed that patients operated on in emergencies had a significantly higher risk of death than those operated on electively (OR = 47) [94].

Peritonitis was ranked third, with a prevalence of 26.5% (95% CI: 22.2–30.9). The etiologies are diverse and are particularly dominated by complicated appendicitis, ranging from 35.7 to 25.5% [95, 96]. With the improvement in medical treatment, including antibiotics and proton pump inhibitors, perforations due to gastroduodenal ulcers and typhoid fever remain less frequent as causes of peritonitis [97, 98]. The occurrence of complications mainly depends on the promptness of initiating treatment [97].

Traumas have a significant impact in terms of frequency on healthcare systems. In our review, abdominal traumas (penetrating and blunt) had a combined prevalence of 9.4% (95% CI: 7.5–11.3). The relatively lower prevalence of abdominal trauma among abdominal emergency surgeries, ranking 6th, may be attributed to advancements in nonoperative management. This leads to fewer surgical interventions due to trauma in abdominal surgical emergency cohorts. However, it is crucial to identify the factors influencing the outcomes of abdominal trauma in Africa to enhance patient care and minimize morbidity and mortality rates [99, 100].

The combined overall mortality was 7.4% (95% CI: 6.0-8.8) in our literature review. Patients undergoing abdominal emergency surgery, when compared to other patients receiving elective surgery, have a higher risk of death (up to 5 times) [4]. In addition, mortality related to surgical emergencies remains consistently high in the world’s poorest countries, where it is estimated to be between 4.9% and 13.2% [4,5,9]. To improve both the availability and quality of treatments, it is essential to have a detailed understanding of the treatments and associated outcomes for patients, particularly operative mortality. The Lancet Commission on Global Surgery recommends the compulsory assessment of postoperative mortality in all healthcare facilities by 2030 as one of the six measures to evaluate the safety of a country’s surgical system [1, 101]. Analyzing factors related to early postoperative death would enable preventive measures to better plan treatment and postoperative outcomes.

The overall morbidity was 24.2% (95% CI: 19.4–19.0) in abdominal surgical emergencies in Sub-Saharan Africa. This high rate is particularly associated with the occurrence of postoperative infections, which are the main complication with an estimated frequency of 14.4% (95% CI: 10.86–18.06). The high rate of complications could be mainly related to a lack of access to surgical care when needed. In fact, financial and geographic components have previously been identified as barriers in Sub-Saharan Africa [10, 102].

### Limitations and perspectives

This review primarily focused on published data collected from databases and excluded unpublished studies such as dissertations or reports, as well as certain data from nonindexed databases or paper-based journals. This exclusion may result in publication bias, as shown by funnel plots with statistically significant Kendall tau tests. There is also a need for improvement and standardization in future studies to elevate the overall level of evidence and effectively guide health system policies.Furthermore, the review did not investigate additional important topics, such as the cost of surgical procedures and patient satisfaction.

The I^2^ test revealed significant heterogeneity, indicating that the included studies may not be comparable when considering design, sample size, patient characteristics, and follow-up. More than half of the included studies were retrospective, single-center studies. Also, due to the non-indexed nature of most articles, it is possible that some published articles were not identified in our search.

As a perspective, given that our review revealed a non-negligible prevalence of death and complications in abdominal surgical emergencies, future studies should be conducted with a more specific focus on exploring the factors associated with mortality and complications in abdominal surgical emergencies. This emphasis on understanding these factors can contribute to the development of preventive measures in health systems and patient management, aiming to effectively reduce the overall rate of deaths in such cases.

Despite these limitations, our review provides significant insights and an overall perspective on the state of research and practice in abdominal emergency surgery in Sub-Saharan Africa. This knowledge can be used to direct future research efforts and to improve the quality of care and identify areas for further improvement.

## Conclusion

Our study suggested a high prevalence of complications, such as deaths and infections. Appendicitis is the main cause of these surgical emergencies. Therefore, it is essential to make efforts to improve access to and quality of patient care. With the relatively low quality of data and heterogeneity of studies, more research is needed to fully understand the quality of care and outcomes for patients undergoing these procedures.

## Data Availability

Data that support the findings of this study are available from the corresponding author upon reasonable request.
